# Uveitis in Patients with Inflammatory Bowel Disease: Pharmacovigilance Signal Detection and Nationwide Claims-Based Evaluation

**DOI:** 10.3390/jcm15218291

**Published:** 2026-10-24

**Authors:** Sunjin Hwang, Jiyeong Kim, Eun Hee Hong, Jee-Eun Chung, Seong Joon Ahn

**Affiliations:** 1 Department of Ophthalmology, Hanyang University Guri Hospital, Guri 11923, Republic of Korea; sunjin1989@hanyang.ac.kr (S.H.); ehhong@hanyang.ac.kr (E.H.H.); 2 Department of Pre-Medicine, College of Medicine, and Biostatistics Laboratory, Medical Research Collaborating Center, Hanyang University, Seoul 04763, Republic of Korea; kimzi@hanyang.ac.kr; 3 College of Pharmacy, Hanyang University, Ansan 15588, Republic of Korea; 4 Department of Ophthalmology, Hanyang University Hospital, Hanyang University College of Medicine, Seoul 04763, Republic of Korea

**Keywords:** uveitis, vedolizumab, ustekinumab, adalimumab, inflammatory bowel diseases

## Abstract

**Background/Objectives**: To evaluate uveitis risk among biologic users with IBD using signal detection and nationwide outcome data. **Methods**: We performed a disproportionality analysis of the FDA Adverse Event Reporting System and a retrospective nationwide cohort study of 7862 Korean patients with IBD who initiated adalimumab, vedolizumab, or ustekinumab between 2015 and 2024. Incident uveitis, identified by diagnostic codes, was compared before and after biologic initiation in exploratory analyses to calculate incidence rate ratios, with post-exposure divided into 0–90 and beyond 90 days; cumulative incidence and multivariable Cox regression were also assessed. Post-switch uveitis risk was compared between patients switching from adalimumab to vedolizumab or ustekinumab. **Results**: Disproportionality analysis identified positive signals for adalimumab (reporting odds ratio, 4.97) and vedolizumab (2.80), but not ustekinumab (0.63). No statistically significant increase in uveitis incidence was observed after biologic initiation in the cohort. No events occurred within 90 days of vedolizumab or ustekinumab initiation; beyond 90 days, incidence rate ratios were 1.3 (95% confidence interval [CI], 0.6–2.9) and 0.6 (95% CI, 0.2–1.7). Adjusted cumulative incidence at the end of follow-up was 1.1%, 0.6%, and 0.2% for adalimumab, vedolizumab, and ustekinumab, with no significant differences on Cox regression. Post-switch risk was similar between vedolizumab and ustekinumab (hazard ratio, 0.88; 95% CI, 0.14–5.58). **Conclusions**: Pharmacovigilance signals for adalimumab and vedolizumab were not reproduced in a nationwide cohort, in which no statistically significant difference in uveitis risk was detected among the three biologics. Given the small number of events and potential selection bias, these descriptive findings do not exclude clinically important differences between treatments.

## 1. Introduction

Inflammatory bowel disease (IBD), including ulcerative colitis (UC) and Crohn’s disease (CD), is a chronic immune-mediated disorder that can frequently be accompanied by extraintestinal manifestations (EIMs) [1,2,3,4]. These EIMs can involve multiple organ systems, including the musculoskeletal and dermatologic systems, as well as the eyes. Although ocular EIMs are less common overall, uveitis is one of the most frequent and clinically important ocular manifestations [5,6].

Biologic therapies are the cornerstone of treatment for moderate-to-severe IBD [7,8,9,10,11]. Tumor necrosis factor (TNF) inhibitors, such as adalimumab, have long been established as effective therapies for both intestinal inflammation and certain EIMs, including noninfectious uveitis. More recently, biologics with distinct mechanisms of action, including vedolizumab, an α4β7 integrin antagonist, and ustekinumab, an interleukin (IL)-12/23 antagonist, have expanded treatment options for patients with IBD [7,8,12,13]. However, because these agents differ in their immunologic targets and tissue selectivity, their effects on ocular inflammation may not be equivalent [3,14,15].

Emerging evidence has raised concerns that biologic therapies may be associated with paradoxical or new-onset extraintestinal inflammatory events, including uveitis [14,15,16,17,18]. Pharmacovigilance analyses have identified uveitis signals for several immunomodulatory agents [19], while case reports and real-world studies have described ocular inflammation and other EIMs during treatment with biologics, particularly vedolizumab [14,15]. However, spontaneous reporting systems are limited by reporting bias, lack of denominator data, and confounding by indication, making it difficult to determine whether the observed associations reflect true drug-related risk [20]. Moreover, comparative population-based data evaluating the risk of uveitis across commonly used biologic therapies for IBD remain limited.

In this study, we evaluated pharmacovigilance signals for uveitis associated with adalimumab, vedolizumab, and ustekinumab using the FDA Adverse Event Reporting System (FAERS) and assessed the risk of incident uveitis after initiation of each biologic using a nationwide South Korean claims database. To complement between-drug comparisons and reduce the influence of time-invariant between-person differences, we employed two additional within-person comparisons: (1) comparisons of pre-exposure and post-exposure uveitis incidence among new users of each biologic and (2) analyses of patients who switched from adalimumab to vedolizumab or ustekinumab, including direct post-switch comparisons between the two therapies with adjustment for clinical covariates.

## 2. Materials and Methods

### 2.1. FAERS Disproportionality Analysis

FAERS disproportionality analyses were performed to evaluate pharmacovigilance signals for uveitis associated with biologics used for IBD. These analyses were intended for signal detection rather than estimation of incidence or causal inference [20]. We extracted FAERS reports through 31 December 2025 and identified reports listing any of seven biologics used for IBD (adalimumab, golimumab, infliximab, natalizumab, risankizumab, ustekinumab, and vedolizumab) as the primary suspect drug. Although the nationwide cohort analysis was limited to adalimumab, vedolizumab, and ustekinumab, all seven biologics were included to place the signals for these three agents in the context of other biologics used for IBD. Duplicate reports were removed by retaining the most recent version of each case (CASEID), based on the latest FDA receipt date and the highest PRIMARYID, and reports from the literature or clinical studies were excluded (Supplementary Figure S1). Uveitis events were identified using the Medical Dictionary for Regulatory Activities (MedDRA) preferred terms (PTs) listed in Supplementary Table S1; the number of reported uveitis events for each biologic was summarized. Because some PTs could be clearly classified as infectious or noninfectious uveitis, while others could belong to both categories, we pre-specified a PT grouping strategy into infectious, noninfectious, or both, and analyses were repeated separately for infectious and noninfectious PTs, with PTs classified as both included in both analyses (Supplementary Tables S2 and S3). Disproportionality was assessed using the reporting odds ratio (ROR), information component (IC), and empirical Bayes geometric mean (EBGM), which compare the observed vs. expected reporting frequency of uveitis for each drug with that in all other reports in the deduplicated database (*n* = 15,194,241), regardless of indication [21]. A positive signal was defined as at least 3 reported cases with a lower limit of the 95% confidence interval (CI) of the ROR (ROR025) above 1 and a lower limit of the 95% credibility interval of the IC (IC025) above 0, consistent with commonly used thresholds for signal detection in spontaneous reporting systems [22,23]. EBGM and its lower 5th percentile (EBGM05) were additionally reported as a supportive Bayesian measure of disproportionality. Three sensitivity analyses were performed: (1) exclusion of 16,755 reports in which uveitis was listed as an indication (*n* = 15,177,486; Supplementary Table S4); (2) restriction of the event definition strictly to the single Preferred Term “Uveitis”, excluding broader terms such as eye inflammation, scleritis, and Behçet’s syndrome (Supplementary Tables S1 and S5); and (3) restriction to reports listing Crohn’s disease or ulcerative colitis as the indication, with all other reports in this subset used as the background (*n* = 368,256; Supplementary Table S6).

### 2.2. Study Design and Population

We conducted a retrospective nationwide cohort study using population-based health insurance claims data. This design is well-suited for evaluating associations between drug exposure and clinical outcomes, particularly when exposures or outcomes are uncommon, and a large population is needed to achieve adequate statistical power [24,25]. In addition, the longitudinal structure of claims data enables clear temporal ordering between biologic initiation and subsequent uveitis onset.

The primary data source was the Health Insurance Review and Assessment (HIRA) database, a comprehensive claims database that covers approximately 98% of the South Korean population [26]. HIRA contains detailed information on inpatient and outpatient services, including demographics (e.g., age and sex), diagnoses coded using the Korean Classification of Diseases (KCD-8, based on ICD-10), procedures, and prescription records [27]. We used de-identified, patient-level longitudinal data, which allow the linkage of individual records across multiple years for time-to-event (TTE) analyses. The nationwide coverage and longitudinal patient-level data enabled evaluation of rare outcomes in routine clinical practice [22,28].

**Figure 1:**
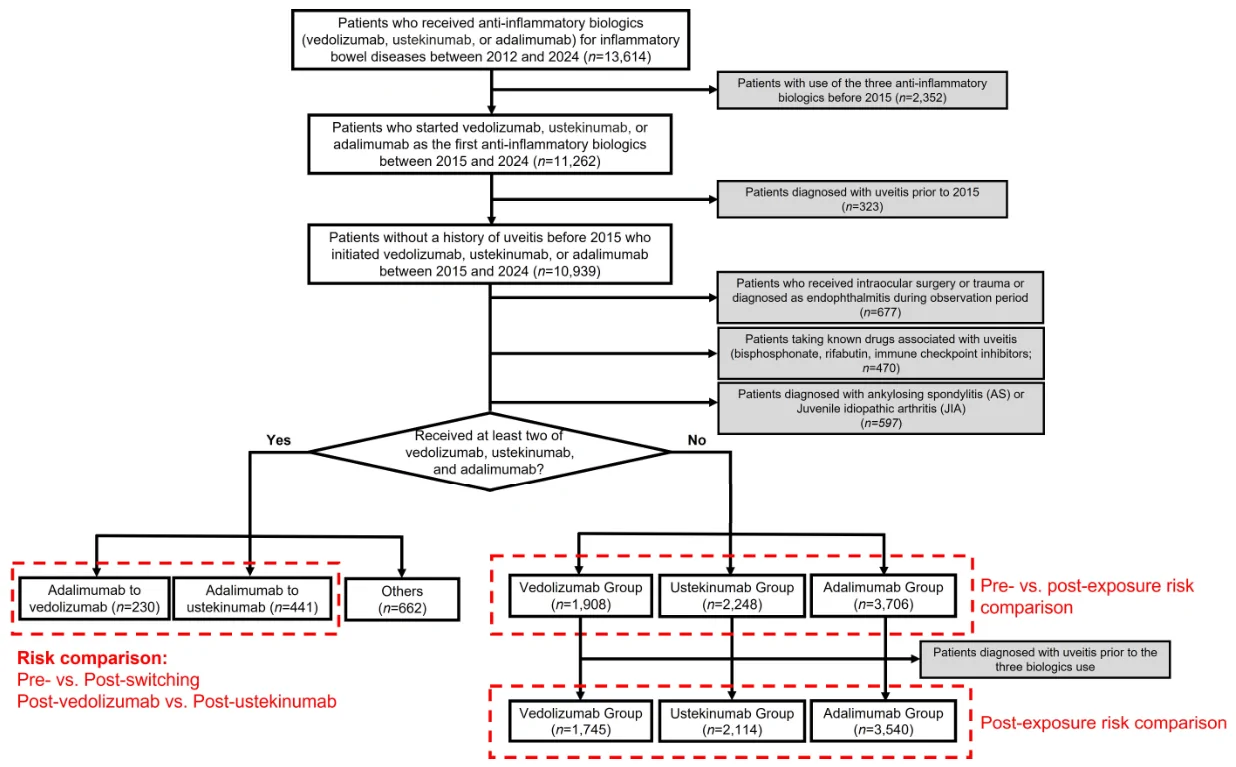
Flowchart illustrating the study design, inclusion and exclusion criteria, and the number of participants.

The study period spanned from 1 January 2012 to 31 December 2024. After applying a washout period from 2012 to 2014, we included patients with UC or CD who were newly initiated on adalimumab, vedolizumab, or ustekinumab between 1 January 2015, and 31 December 2024. IBD was defined by the KCD-8 codes K50 (CD) and K51 (UC); the rare intractable disease registration codes were not used. Exposure to adalimumab included both the originator and biosimilar products. The cohort analysis was prespecified to compare a single anti-TNF reference agent with biologics of different mechanistic classes, namely vedolizumab, a gut-selective anti-integrin agent, and ustekinumab, an anti-IL-12/23 agent. Adalimumab was selected as the anti-TNF reference because it is the only biologic approved for noninfectious uveitis; therefore, other anti-TNF agents, including infliximab, were not included in the cohort analysis. Patients with any recorded diagnosis of uveitis before 2015 were excluded from the study. We also excluded patients who had undergone intraocular surgery or ocular trauma, or who were diagnosed with endophthalmitis, at any time during the study period, including after the index date (Supplementary Table S7) and those exposed to drugs known to be associated with uveitis at any time during the study period (e.g., bisphosphonates, rifabutin, or immune checkpoint inhibitors; Supplementary Table S8). To reduce confounding by systemic rheumatologic diseases that are strongly linked to uveitis, we excluded patients diagnosed with ankylosing spondylitis or juvenile idiopathic arthritis. The patient selection is summarized in Figure 1. 

This study adhered to the principles of the Declaration of Helsinki. The Institutional Review Board of Hanyang University Hospital approved this study (IRB No. 2025–09–011; approval date, 25 September 2025) and waived the need for informed consent because only de-identified data were used. This study was reported in accordance with the READUS-PV guideline for disproportionality analyses and the STROBE guideline for observational studies; the completed checklists are provided in the Supplementary Materials.

### 2.3. Study Outcomes and Definitions

The index date for each participant was defined as the date of their first biologic prescription. Exposure was assigned according to the first study biologic initiated (index biologic). A pre-exposure period, spanning from the start of the available data (1 January 2012) to the index date, was defined for each patient to enable pre-exposure vs. post-exposure comparisons within individuals. Because patients with uveitis diagnosed before 1 January 2015, were excluded, the 2012–2014 portion of the pre-exposure period contributed person-time but no events. Accordingly, the length of the pre-exposure period varied with the date of biologic initiation. Patients who received two or more of the three study biologics during the study period were not included in the main cohort and were analyzed separately in the switcher analyses; therefore, no person-time after switching between study biologics was attributed to the index biologic. Participants were followed from the index date until the first uveitis event, death, or the end of the study period (31 December 2024), whichever occurred first. Consistent with an initial-treatment (as-started) approach, follow-up was not censored at treatment discontinuation, because discontinuation dates cannot be reliably determined from claims data for biologics administered at intervals of up to 8 to 12 weeks, and because uveitis may develop after a delay. The interval from biologic initiation to the first documented uveitis event was assessed, and the post-exposure time was segmented into two risk windows: 0–90 and >90 days.

The primary outcome was incident uveitis, which was identified using KCD-8 diagnostic codes derived from the ICD-10 (Supplementary Table S9). Overall, uveitis and subtype-specific outcomes were classified as anterior vs. non-anterior uveitis based on anatomical location, consistent with the classification approach used in prior uveitis studies using the same database [28,29]. To improve diagnostic reliability, all uveitis outcomes required the relevant KCD-8 code to appear on at least two separate claims, and a visit to an ophthalmology department was not required. The date of the first claim was used as the event date. Codes specific to infectious uveitis (e.g., tuberculous, toxoplasma, or herpetic ocular infection) were not included in the outcome definition, so infectious uveitis could not be identified separately, and uveitis coded with nonspecific codes may include some infectious cases.

### 2.4. Switcher Analyses

As a secondary analysis, we evaluated patients who switched biologic therapy to reduce the influence of time-invariant between-person differences. Because switching from adalimumab to either vedolizumab or ustekinumab represented the majority of switching patterns, analyses were restricted to these two prespecified sequences. Two comparisons were conducted: (1) a within-person pre-switch vs. post-switch comparison of uveitis incidence, wherein each patient’s adalimumab exposure period served as an internal control for the subsequent vedolizumab or ustekinumab periods and (2) a between-group comparison of post-switch uveitis risk between vedolizumab and ustekinumab using an age-adjusted Cox proportional hazards model. Because only five events occurred after switching, adjustment for additional covariates was not feasible, and this comparison was considered exploratory. In the switcher analyses, person-time before switching was classified as adalimumab exposure and person-time after switching as post-switch exposure, beginning on the first prescription date of the new biologic. The follow-up time was censored at the time of uveitis occurrence, switching to an additional biologic, death, or end of the study period (31 December 2024), whichever occurred first.

### 2.5. Statistical Analyses

For each biologic group, we compared the uveitis incidence rate (IR) during the post-exposure risk windows with that during the pre-exposure period among the same patients. IRs were calculated per person-year (PYs), and crude incidence rate ratios (IRRs) with 95% confidence intervals (CIs) were estimated. The IRRs were not adjusted for age, calendar time, or secular trends in uveitis coding. Because each patient contributed person-time to both periods, this comparison reduces the influence of time-invariant patient characteristics. However, it is not a formal self-controlled design, such as the self-controlled case series, and it does not account for time-varying factors, such as disease activity, corticosteroid treatment, or treatment escalation, that may change around the time of biologic initiation. Patients with a uveitis event in the pre-exposure period remained in the post-exposure follow-up; therefore, post-exposure events in these patients may represent recurrence rather than new-onset uveitis. Because the event-free 2012–2014 period was included in the pre-exposure person-time, the pre-exposure IRs were likely underestimated and the IRRs overestimated, and the magnitude of this bias may have differed between biologic groups with different distributions of initiation dates. The pre- vs. post-exposure comparisons were therefore considered descriptive and exploratory.

To describe the clinical context of each treatment group and assess the potential between-group differences at initiation, we summarized and compared baseline characteristics across the three biologic groups. Continuous variables were compared using one-way analysis of variance (ANOVA), and categorical variables were compared using the chi-square test or Fisher’s exact test. Baseline variables included age, sex, IBD subtype (UC and CD), prescribing specialty, systemic comorbidities (hypertension, diabetes, dyslipidemia, liver diseases, and chronic kidney diseases), baseline EIMs other than uveitis (musculoskeletal, dermatologic, hepatopancreatobiliary, and other ophthalmic diseases), and baseline systemic corticosteroid use.

For the between-group comparisons of post-exposure risk, patients diagnosed with uveitis between 1 January 2015, and the index date were additionally excluded, so that these analyses evaluated incident uveitis after biologic initiation in patients without previous uveitis (vedolizumab, *n* = 1745; ustekinumab, *n* = 2114; adalimumab, *n* = 3540; Figure 1). The pre- vs. post-exposure comparisons included all 7862 patients, because the pre-exposure period served as the reference. Kaplan–Meier curves were generated to illustrate the time to the first occurrence of uveitis by subtype. Cumulative incidence at 1 year and until the end of the study was calculated using life-table methods. Age- and sex-adjusted cumulative incidence was obtained by direct standardization, weighting the stratum-specific cumulative incidence by the age and sex distribution of the total cohort. Cox proportional hazards models were used to compare the time to first uveitis between biologic groups. Candidate covariates were age, sex, IBD subtype, systemic comorbidities (diabetes mellitus, hypertension, dyslipidemia, liver diseases, and chronic kidney diseases), and baseline systemic corticosteroid use. Because the number of uveitis events was small, a parsimonious multivariable model was constructed to limit overfitting: biologic group was forced into the model, and candidate covariates with *p* < 0.05 in univariable analyses were included. The proportional hazards assumption was evaluated by visual inspection of the Kaplan–Meier curves. Adalimumab was used as the reference group because of its established efficacy for noninfectious uveitis.

All analyses were two-sided, with *p* < 0.05 considered statistically significant. Statistical analyses were performed using R version 4.3.2 (R Foundation for Statistical Computing, Vienna, Austria) and SAS Enterprise Guide Software 7.1 (SAS Institute Inc., Cary, NC, USA).

## 3. Results

### 3.1. Pharmacovigilance Signals for Uveitis Across Biologic Therapies (Supplementary Figure S1)

Table 1 summarizes uveitis-related adverse event reports in the FAERS database for each biologic, regardless of the reported indication. Infliximab showed the strongest disproportionality signal among all biologics evaluated (*n* = 1896; reporting odds ratio [ROR], 11.98; 95% CI, 11.42–12.56; IC, 3.45; EBGM, 10.91), followed by adalimumab, which had the greatest number of reports (*n* = 2986; ROR, 4.97; 95% CI, 4.78–5.17; IC, 2.15; EBGM, 4.43). Vedolizumab demonstrated positive disproportionality, with more than 200 reports (*n* = 289; ROR, 2.80; 95% CI, 2.49–3.15; IC, 1.47; EBGM, 2.77). Golimumab also demonstrated positive disproportionality (*n* = 204; ROR, 3.11; 95% CI, 2.71–3.57; IC, 1.62; EBGM, 3.08). Ustekinumab, however, showed no evidence of positive disproportionality (*n* = 66; ROR, 0.63; 95% CI, 0.49–0.80; IC, −0.67; EBGM, 0.63), and neither did risankizumab (*n* = 52; ROR, 0.47; 95% CI, 0.36–0.62) or natalizumab (*n* = 42; ROR, 0.46; 95% CI, 0.34–0.63); for these three agents, the upper limit of the 95% CI of the ROR was below 1. The results were similar when infectious and noninfectious PTs were analyzed separately (Supplementary Tables S2 and S3).

In sensitivity analyses, positive signals persisted for infliximab (ROR, 11.53; 95% CI, 10.96–12.14), adalimumab (ROR, 3.42; 95% CI, 3.26–3.59), golimumab (ROR, 3.41; 95% CI, 2.96–3.92), and vedolizumab (ROR, 3.12; 95% CI, 2.78–3.51) after exclusion of reports in which uveitis was listed as an indication (Supplementary Table S4). Restricting the event definition to narrow uveitis-specific PT did not remove any positive signal; the RORs were higher than in the main analysis for infliximab (23.16), adalimumab (7.05), golimumab (5.00), and vedolizumab (4.00), whereas the ROR for ustekinumab moved toward 1 (0.95; 95% CI, 0.71–1.27) (Supplementary Table S5). In contrast, when the analysis was restricted to reports listing Crohn’s disease or ulcerative colitis as the indication, adalimumab no longer showed a positive signal (*n* = 402; ROR, 0.41; 95% CI, 0.37–0.46), whereas positive signals remained only for infliximab (*n* = 1250; ROR, 5.87; 95% CI, 5.39–6.40). Golimumab, ustekinumab, vedolizumab, and risankizumab showed no positive signal, and no uveitis reports were identified for natalizumab in this subset (Supplementary Table S6). These estimates reflect disproportionate reporting relative to all other drugs in FAERS and do not represent the incidence of uveitis or a causal drug effect.

### 3.2. Baseline Characteristics and Patterns of Nationwide Biologic Use in Patients with IBD

Between 2012 and 2024, 13,614 patients with IBD received at least one of the three biologic agents. After applying the washout period and excluding uveitis diagnoses before 2015, 10,939 patients were enrolled in the primary analytic cohort. After further exclusions (Figure 1), the final analytic cohort comprised 7862 patients with IBD who were new users of the studied biologics: vedolizumab (*n* = 1908), ustekinumab (*n* = 2248), and adalimumab (*n* = 3706).

Baseline characteristics differed across the biologic groups (Table 2). Vedolizumab initiators were the oldest (mean 40.3 ± 15.6 years), followed by ustekinumab (35.2 ± 13.4) and adalimumab initiators (30.0 ± 14.0) (*p* <0.001). Male patients accounted for roughly two-thirds of each group, with modest but significant differences (*p* = 0.026). Gastroenterologists prescribed most biologics, especially vedolizumab (88.2%) and ustekinumab (84.2%), while 24.7% of adalimumab prescriptions were from other specialties (*p* < 0.001). UC predominated in vedolizumab users (81.6%), whereas CD was more common in adalimumab (63.0%) and ustekinumab (59.2%) users (*p* < 0.001). Vedolizumab users had higher rates of diabetes, hypertension, and dyslipidemia than adalimumab and ustekinumab users (all *p* < 0.001). Baseline extraintestinal manifestations other than uveitis and systemic corticosteroid use (highest in vedolizumab, 51.6%) also varied across the groups (*p* < 0.001).

**Figure 2:**
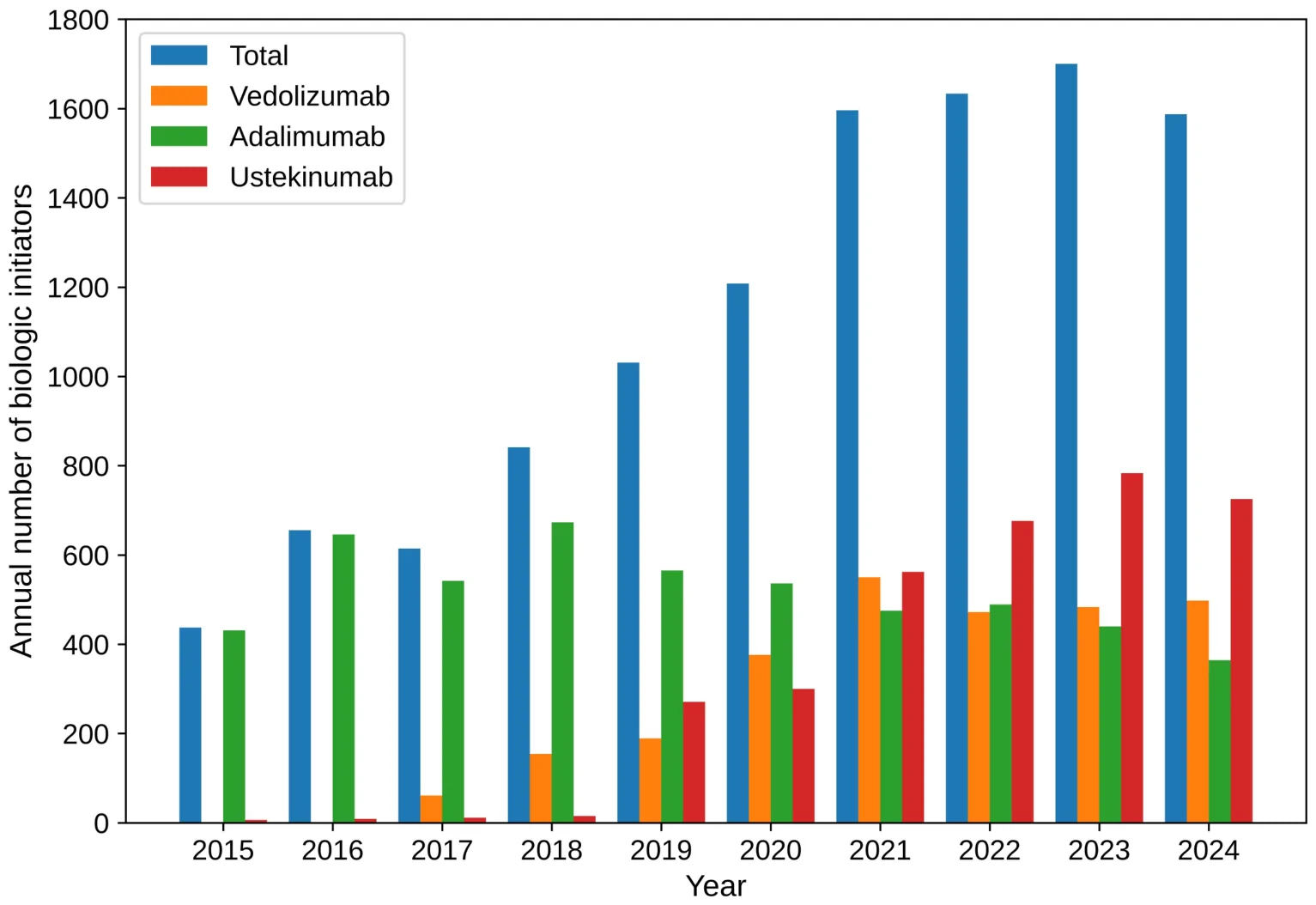
Annual number of patients with inflammatory bowel disease (ulcerative colitis [UC] and Crohn’s disease [CD]) and of those treated with vedolizumab, ustekinumab, and adalimumab in South Korea between 2015 and 2024.

Across the study period, the annual number of biologic initiators increased from 433 in 2015 to 1589 in 2024 (Figure 2). Adalimumab was the most frequently initiated biologic early in the study, accounting for 429 of 433 initiations in 2015; vedolizumab use began in 2017 and steadily increased thereafter. Ustekinumab use increased substantially from 2019 onward, reaching 730 initiations in 2024. Vedolizumab initiations grew rapidly from 61 in 2017 to 552 in 2021 and stabilized at 483–496 per year by 2023–2024. Meanwhile, adalimumab initiations declined gradually after peaking in 2018 (671 initiations) to 363 in 2024.

### 3.3. IRRs of Uveitis Before and After Biologic Exposure

Uveitis events were rare across all biologic groups, resulting in wide CIs for several estimates. Because the pre-exposure IRs were likely underestimated, these comparisons are descriptive and exploratory. Table 3 summarizes uveitis incidence rates (IRs) during the pre-exposure period and the overall post-exposure period, as well as across post-exposure risk windows (0–90 and >90 days), for each biologic, stratified by anatomic subtype (anterior vs. non-anterior). For vedolizumab, during the overall post-exposure period, the IR for overall uveitis was 14.7 per 10,000 PYs, corresponding to an IRR of 1.1 (95% CI, 0.5–2.6) compared with the pre-exposure period. No uveitis events occurred within the first 90 days (IR, 0.0 per 10,000 PYs). Beyond 90 days, the IR increased to 16.3 per 10,000 PYs (IRR, 1.3; 95% CI, 0.6–2.9). Subtype analyses showed that anterior uveitis (IRR, 1.0; 95% CI, 0.4–2.6 in the overall post-exposure period; IRR, 1.1; 95% CI, 0.4–2.9 beyond 90 days) accounted for most events, while non-anterior uveitis remained infrequent (IRR, 1.8; 95% CI, 0.4–9.4 overall; IRR, 2.0; 95% CI, 0.4–10.5 beyond 90 days).

For adalimumab, during the overall post-exposure period, the IR for overall uveitis was 15.0 per 10,000 PYs (IRR, 0.8; 95% CI, 0.5–1.3) compared with the pre-adalimumab period. Within 0–90 days, four uveitis events occurred (IR, 44.1 per 10,000 PYs), yielding an IRR of 2.4 (95% CI, 0.9–6.5). After 90 days, the IR decreased to 13.6 per 10,000 PYs (IRR, 0.7; 95% CI, 0.5–1.2). A similar pattern was observed for anterior uveitis, while non-anterior uveitis events were rare across periods.

For ustekinumab, during the overall post-exposure period, the IR for overall uveitis was 7.6 per 10,000 PYs, corresponding to an IRR of 0.5 (95% CI, 0.2–1.5) compared with the pre-exposure period. No uveitis events were observed within 90 days (IR, 0.0 per 10,000 PYs). Beyond 90 days, the IR was 8.5 per 10,000 PYs (IRR, 0.6; 95% CI, 0.2–1.7). All observed events were anterior uveitis, with no cases of non-anterior uveitis in the post-exposure period.

Stratified analyses by IBD subtype showed similar patterns for both ulcerative colitis (Supplementary Table S10) and Crohn’s disease (Supplementary Table S11), with no statistically significant differences in IRRs between pre-exposure and overall post-exposure periods across biologics. However, in patients with ulcerative colitis, the IR within 90 days after adalimumab initiation was significantly higher than that in the pre-exposure period (89.6 vs. 24.5 per 10,000 PYs; IRR, 3.7; 95% CI, 1.1–12.0), although this estimate was based on three events and is likely to be overestimated. This early increase was not observed in patients with Crohn’s disease (IRR, 1.1; 95% CI, 0.2–8.3), and it was not statistically significant in the overall cohort (IRR, 2.4; 95% CI, 0.9–6.5; Table 3).

### 3.4. Cumulative Incidence of Uveitis After Biologic Exposure

Among the 7399 patients without uveitis before biologic initiation, the cumulative incidence of overall uveitis at 1 year was 0.25% for vedolizumab, 0.17% for adalimumab, and 0.05% for ustekinumab (Table 4); by the end of follow-up, the cumulative incidence increased to 0.61%, 1.29%, and 0.33%, respectively. After adjusting for age and sex, the 1-year incidence rates were 0.23% for vedolizumab, 0.15% for adalimumab, and 0.03% for ustekinumab, reaching 0.58%, 1.14%, and 0.17%, respectively, by study end. For anterior uveitis, the cumulative incidence at 1 year was 0.19% for vedolizumab, 0.17% for adalimumab, and 0.05% for ustekinumab, which increased to 0.45%, 1.16%, and 0.33%, respectively, at the end of follow-up. Non-anterior uveitis accounted for a substantially smaller proportion of events, with cumulative incidences of 0.16%, 0.13%, and <0.01% at the end of the study for the vedolizumab, adalimumab, and ustekinumab groups, respectively. The cumulative incidence curves over time are presented in Supplementary Figure S2.

### 3.5. Association of Biologic Therapy with Incident Uveitis in Patients with IBD

In the Cox proportional hazards models for overall uveitis (Table 5), neither vedolizumab nor ustekinumab showed a significant difference in risk compared with adalimumab after multivariable adjustment for IBD subtype, hypertension, and baseline systemic corticosteroid use (vedolizumab vs adalimumab: HR, 0.67; 95% CI, 0.28–1.62; ustekinumab vs adalimumab: HR, 0.53; 95% CI, 0.18–1.54), although the CIs were wide. UC (vs CD) was associated with increased risk in univariable analysis (HR, 2.26; 95% CI, 1.18–4.34), but this association was no longer statistically significant after adjustment (HR, 1.86; 95% CI, 0.92–3.73, *p* = 0.083). Baseline systemic corticosteroid use showed a significant association in univariable analysis (HR, 2.05; 95% CI, 1.08–3.90), which was no longer statistically significant in the multivariable model (HR, 1.73; 95% CI, 0.90–3.33, *p* = 0.10). Overall, no statistically significant difference in overall uveitis risk was detected between biologics after multivariable adjustment. However, the precision of the estimates was limited, the covariates were selected by univariable screening, and the proportional hazards assumption was not formally tested; these adjusted HRs therefore do not provide robust evidence of comparative treatment effects.

### 3.6. Uveitis Risk in Switchers from Adalimumab to Vedolizumab or Ustekinumab

Supplementary Table S12 compares the overall incidence of uveitis before (adalimumab exposure) and after switching among patients switching to vedolizumab or ustekinumab. In the adalimumab-to-vedolizumab group, no uveitis events were observed during the adalimumab-exposure period; however, two events occurred after switching to vedolizumab (IR, 18.9 per 10,000 person-years), precluding the estimation of the IRR. In the adalimumab-to-ustekinumab group, two events occurred during the adalimumab-exposure period and three events occurred after switching to ustekinumab, yielding an IR of 13.1 and 24.2 per 10,000 PYs, respectively, with an IRR of 1.9 (95% CI, 0.3–11.1). In the age-adjusted Cox model comparing the post-switch periods, the risk of uveitis did not differ significantly between the vedolizumab and ustekinumab groups (HR, 0.88; 95% CI, 0.14–5.58).

## 4. Discussion

In this nationwide cohort study, we evaluated uveitis risk among patients with IBD receiving commonly used biologic therapies using complementary pharmacovigilance and population-based approaches. Although FAERS disproportionality analyses identified positive uveitis signals for adalimumab and vedolizumab, these signals were not reproduced in the nationwide claims-based cohort. In the between-group comparisons, we did not detect statistically significant differences in uveitis risk among adalimumab, vedolizumab, and ustekinumab. Uveitis events were uncommon across all treatment groups, and in exploratory pre- vs. post-exposure comparisons, no statistically significant increase in risk was observed after initiation of any biologic. However, because the number of events was small, particularly for vedolizumab and ustekinumab, several estimates had wide CIs, and these findings should not be interpreted as evidence of equivalent risk. In addition, the cohort analyses could not distinguish the effects of treatment from those of underlying disease activity or differences in patient selection, and several potential sources of bias could not be addressed. The findings are therefore best regarded as descriptive population-based observations that complement the pharmacovigilance signals, rather than as estimates of comparative safety.

The biologic therapies evaluated in this study differ substantially in their mechanisms of immune modulation, which may theoretically influence ocular inflammatory outcomes. Vedolizumab selectively blocks α4β7 integrin-mediated lymphocyte trafficking to the gut, raising the possibility that its gut-selective mechanism may provide less systemic control of extraintestinal inflammation compared with agents such as adalimumab [8,12,13]. In a retrospective single-center study by Diaz et al., de novo EIMs occurred in 19 of 71 (26.7%) vedolizumab-treated patients after a median of 3.75 months; however, uveitis accounted for only 1 (1.41%) case, indicating that uveitis was uncommon even in a cohort assembled specifically to examine new EIMs [14]. Dubinsky et al. reported that patients with CD treated with vedolizumab were 28% more likely to develop any extraintestinal manifestations (EIMs) overall compared with those treated with anti-TNF agents (adjusted IRR, 1.28; 95% CI, 1.02–1.62) [30]. Furthermore, uveitis/iritis was among the EIMs more frequently observed in vedolizumab-treated patients, with an IRR of 2.89 (95% CI, 1.35–6.18) [30]. Conversely, adalimumab has demonstrated efficacy in noninfectious uveitis and may theoretically reduce ocular inflammatory activity through systemic TNF inhibition [9,10]. Ustekinumab, which targets the IL-12/23 pathway, provides an additional non-TNF comparator with a distinct immunologic mechanism [11,16,17]. Despite these mechanistic differences, and although FAERS analyses identified positive uveitis signals for adalimumab and vedolizumab, we did not detect statistically significant differences in uveitis risk among these biologics in the population-based cohort, with similar patterns observed across the UC and CD subgroups (Supplementary Tables S10 and S11). Given the limited precision of these estimates, clinically relevant differences cannot be excluded.

The contrast between our nationwide claims-based findings and the positive FAERS disproportionality signals for vedolizumab likely arose from methodological differences between the two approaches. The two analyses also measure different quantities: disproportionality in FAERS reflects relative reporting across all indications worldwide, whereas the cohort estimates reflect the incidence of uveitis among Korean patients with IBD. Agreement between them should therefore not necessarily be expected. Disproportionality analyses capture reporting imbalances rather than true incidence and, because they lack denominator information for the exposed population, cannot provide unbiased estimates of comparative risk [19]. Regulatory and methodological guidance has consistently emphasized that FAERS should not be used to estimate incidence or directly compare drugs, given its susceptibility to underreporting, differential reporting, missing data, and confounding by co-medication and disease severity [20]. These limitations are particularly relevant, because differential treatment selection in patients with underlying EIM such as uveitis, as well as heightened clinician awareness of potential ocular adverse events, may introduce reporting bias in FAERS, leading to apparent disproportionality signals that do not reflect true differences in incidence. The particularly strong disproportionality signal for infliximab in FAERS should also be interpreted with caution. Infliximab is effective in noninfectious uveitis, with outcomes largely similar to those of adalimumab, and it is frequently used off-label for refractory noninfectious uveitis, including Behcet’s uveitis. Reports in which uveitis was the treated condition, or in which a pre-existing inflammation persisted during treatment, may therefore have been recorded as adverse events. Because infliximab was not included in our prespecified cohort analysis, whether this signal reflects a true increase in risk could not be evaluated in the present study.

The FAERS sensitivity analyses also show how confounding by indication can shape disproportionality signals. In the full database, which includes reports for all indications, adalimumab showed a positive uveitis signal. This signal was attenuated after exclusion of reports listing uveitis as an indication (ROR, 4.97 to 3.42) and was no longer present when the analysis was restricted to reports with Crohn’s disease or ulcerative colitis as the indication (ROR, 0.41; 95% CI, 0.37–0.46). Adalimumab is approved for noninfectious uveitis and is widely used for spondyloarthritis, psoriasis, and juvenile idiopathic arthritis, conditions with a high background risk of uveitis. In the full database, uveitis reports for adalimumab may therefore reflect the underlying disease or the treatment of pre-existing uveitis rather than a drug effect. Similarly, the vedolizumab signal was no longer present in the IBD-restricted analysis, although it persisted in the other sensitivity analyses. Because vedolizumab is used almost exclusively for IBD, its reports are less affected by differences in indication. However, reporting in the main analysis may have been stimulated by published reports of new-onset EIMs during vedolizumab therapy [14,30], and because vedolizumab is often started after failure of anti-TNF therapy, withdrawal of systemic TNF inhibition may unmask previously suppressed ocular inflammation that is then attributed to vedolizumab. The IBD-restricted estimates should also be interpreted with caution, because the indication field in FAERS is frequently incomplete and infliximab accounted for a large proportion of uveitis reports in this subset, which may have lowered the RORs for the other biologics (competition bias). Taken together, the FAERS signals are best interpreted as reflecting reporting patterns, which may differ from the incidence of uveitis observed in the nationwide cohort.

However, baseline clinical heterogeneity among biologic treatment groups in the HIRA cohort should also be considered when interpreting these findings. Patients initiating vedolizumab were older, had more comorbidities, and were more frequently diagnosed with UC compared with those receiving other biologics. These differences are clinically relevant because a recent systematic review and meta-analysis including 115,854 patients with IBD reported that uveitis was approximately twice as prevalent in patients with CD compared with UC (pooled prevalence, 3.3% vs. 1.6%) [31]. Based on these underlying disease characteristics, one might expect lower uveitis incidence among vedolizumab users than among adalimumab users. However, no statistically significant difference in uveitis risk was detected between biologic groups after multivariable adjustment. Because the multivariable model was based on few events, the covariates were selected by univariable screening rather than prespecified, and the proportional hazards assumption was not formally tested, these adjusted estimates do not provide robust evidence of comparative treatment effects, and residual confounding from baseline disease characteristics or unmeasured clinical factors may influence comparative risk estimates cannot be excluded. In addition, hypertension was associated with uveitis risk in the multivariable model (HR, 2.14; 95% CI, 1.07–4.28). Because this association is not well supported biologically and nine candidate covariates were examined without correction for multiple comparisons, it may reflect residual confounding by age or comorbidity burden, more frequent healthcare contact leading to the detection of ocular disease, or chance, and it should not be interpreted as causal. Further studies with more detailed clinical phenotyping and improved control of baseline disease characteristics are needed to validate these findings.

The switcher analysis provided further insight into this interpretation, because within-person comparisons before and after switching may mitigate the influence of fixed patient-level characteristics. Among patients who switched from adalimumab to vedolizumab or ustekinumab, no statistically significant increase in post-switch uveitis was observed in the adalimumab-to-ustekinumab group, and the IRR could not be estimated in the adalimumab-to-vedolizumab group because no events occurred during the adalimumab period. The post-switch risk did not differ significantly between the two post-switch groups. However, these comparisons do not account for time-varying factors, such as disease activity, corticosteroid use, or the clinical reasons for switching, which are likely to differ between the pre- and post-switch periods. Given the small number of events (seven in total) and the wide CIs, these findings are consistent with the overall results but do not exclude a clinically relevant change in uveitis risk after switching to non-TNF agents.

Temporal patterns of events also provide clinical context. In our cohort, no uveitis events occurred within 90 days of initiation among vedolizumab and ustekinumab initiators, and all of their post-initiation events occurred after 90 days. For vedolizumab in particular, this pattern is consistent with the natural history of IBD-associated uveitis, which is often dissociated from luminal disease activity and may occur despite gastrointestinal remission [4,6]. Uveitis has also been associated with other EIM, particularly peripheral arthritis, suggesting that ocular inflammation reflects a broader systemic inflammatory phenotype rather than intestinal activity alone [4,6]. We also hypothesize that, because corticosteroids are a foundational short-term therapy for noninfectious uveitis and steroid tapering is often pursued after intestinal induction, the withdrawal of steroid coverage may allow previously suppressed ocular inflammation to re-emerge over weeks to months. However, this could not be examined in the present study, because corticosteroid dosing and tapering were not analyzed.

In contrast, adalimumab initiators showed a higher IR within the first 90 days than in the pre-exposure period (IRR, 2.4; 95% CI, 0.9–6.5), and this difference was statistically significant among patients with UC (IRR, 3.7; 95% CI, 1.1–12.0). Several explanations should be considered. First, confounding by indication or disease severity is plausible. Because adalimumab is approved for noninfectious uveitis and has long been an established option for IBD with EIMs, it may have been preferentially started in patients with active EIMs or early ocular symptoms. Ocular inflammation that began before initiation may also have been diagnosed or coded only after initiation. Second, detection bias may have contributed, because treatment initiation is accompanied by more frequent clinical contact. Third, a genuine early drug effect, such as a paradoxical inflammatory reaction to TNF inhibition, cannot be excluded, because paradoxical uveitis has been reported during anti-TNF therapy. Finally, the estimate was based on only three events and was one of many IRRs estimated across drugs, risk windows, and uveitis and IBD subtypes, so chance cannot be excluded. In addition, because the pre-exposure rates were likely underestimated, particularly among early initiators, most of whom received adalimumab, this IRR is likely to be overestimated. The absence of an early increase among patients with CD and the lower IR after 90 days than in the pre-exposure period (IRR, 0.7; 95% CI, 0.5–1.2) argue against a sustained drug effect, but our data cannot distinguish among these explanations. Studies with detailed clinical information on ocular symptoms and disease activity at the time of treatment initiation are needed to clarify this finding.

This study has some limitations that should be considered when interpreting the results. First, uveitis events were rare, resulting in wide confidence intervals and limiting the precision of estimates, particularly for vedolizumab and ustekinumab and for non-anterior and other subtype-specific analyses. Based on the number of events, the study had 80% power at a two-sided alpha of 0.05 to detect a hazard ratio of approximately 2.7 or greater (or 0.37 or less) for vedolizumab or ustekinumab compared with adalimumab. Smaller but clinically relevant differences therefore cannot be excluded, and the absence of statistically significant differences should not be interpreted as evidence of equivalent risk. Second, several decisions in the construction of the nationwide cohort used information recorded after biologic initiation and may have introduced selection bias. Patients who received two or more study biologics during follow-up were assigned to the switcher analyses rather than the main cohort, and patients with intraocular surgery, ocular trauma, endophthalmitis, or exposure to uveitis-associated drugs were excluded on the basis of records during the entire study period, including after biologic initiation, rather than censored at the time of these events. Because switching and ocular procedures may be related to disease activity, extraintestinal manifestations, or uveitis itself, and because some intraocular procedures are performed for complications of uveitis, these decisions may have removed patients at higher risk of uveitis, and the extent of this removal may have differed between biologics with different treatment patterns. In addition, exposure was defined by the index biologic using an initial-treatment approach without censoring at discontinuation. Because treatment persistence may differ between biologics, the resulting exposure misclassification may also have differed between groups. The direction and magnitude of these biases cannot be determined, and they limit the validity of the between-group comparisons. Third, we relied on claims-based outcome definitions; individual diagnoses could not be confirmed through a medical record review, and the misclassification of uveitis was possible. Fourth, the pre-exposure period began in 2012, but patients with uveitis before 2015 were excluded. This event-free 2012−2014 period likely led to underestimated pre-exposure rates and overestimated IRRs, and because it made up a larger share of the pre-exposure period in earlier initiators, most of whom received adalimumab, the magnitude of this bias likely differed between groups. The pre-exposure period also varied in length, may have included time before IBD diagnosis, and was not adjusted for age or calendar time. The pre- vs. post-exposure comparisons should therefore be regarded as descriptive and exploratory rather than as estimates of treatment-associated changes in uveitis risk. Fifth, covariates for the Cox models were selected by univariable screening rather than prespecified. With few events, this approach may have omitted important confounders that did not reach statistical significance, and propensity score methods were not used despite baseline imbalances between groups. In addition, the proportional hazards assumption was not formally tested and was assessed only by visual inspection of the Kaplan–Meier curves, which overlapped during the early years of follow-up and diverged later. The adjusted hazard ratios should therefore be interpreted as average associations over follow-up rather than as robust estimates of comparative treatment effects, and the fixed-time cumulative incidence estimates (Table 4) should be considered together. In addition, infliximab, which showed the strongest disproportionality signal in FAERS, was not included in the prespecified cohort analysis, and its signal was therefore not evaluated in population-based data. Moreover, about one-quarter of adalimumab prescriptions were issued by specialties other than gastroenterology or internal medicine. Although this probably reflects the younger age of adalimumab initiators, some adalimumab may have been started for extraintestinal indications, including uveitis, which could have affected the pre- vs. post-exposure comparison. Finally, although our study included a large nationwide cohort, the findings may not be generalizable to other healthcare systems or populations with different prescription patterns or baseline risks. In particular, patients with ankylosing spondylitis or juvenile idiopathic arthritis were excluded to reduce confounding; therefore, our findings may not apply to these patients, who are at the highest risk of uveitis and in whom the choice of biologic may matter most.

## 5. Conclusions

In conclusion, this nationwide cohort study did not detect a statistically significant difference in uveitis risk among patients with IBD treated with adalimumab, vedolizumab, or ustekinumab. Because uveitis events were uncommon, particularly among vedolizumab and ustekinumab users, the estimates were imprecise, and clinically relevant differences cannot be ruled out. The present analyses also could not distinguish the effects of treatment from those of underlying disease activity and differences in patient selection, and several potential sources of bias remain unresolved. Although FAERS analyses identified disproportionate reporting signals for adalimumab and vedolizumab, these signals were not reproduced in the nationwide cohort, and they may reflect reporting patterns, treatment selection, or other underlying differences rather than drug-specific effects. These findings should be regarded as descriptive population-based observations and pharmacovigilance signals rather than evidence on the comparative ocular safety of these biologics. Further studies incorporating validated ophthalmologic outcomes, detailed clinical phenotyping, and designs that account for time-varying disease activity are needed to compare the ocular safety of biologic therapies for IBD.

## Figures and Tables

**Table 1:** Number of reported overall uveitis cases as an adverse event for biologics in the FAERS database (December 2025), listed in descending order by the number of reports.

Rank	Medication	No. of Reports	ROR (95% CI)	IC (IC025)	EBGM (EBGM05)
1	Adalimumab	2986	4.97 (4.78–5.17)	2.15 (2.1)	4.43 (4.29)
2	Infliximab	1896	11.98 (11.42–12.56)	3.45 (3.38)	10.91 (10.48)
3	Vedolizumab	289	2.8 (2.49–3.15)	1.47 (1.3)	2.77 (2.52)
4	Golimumab	204	3.11 (2.71–3.57)	1.62 (1.42)	3.08 (2.74)
5	Ustekinumab	66	0.63 (0.49–0.8)	−0.67 (−1.01)	0.63 (0.51)
6	Risankizumab	52	0.47 (0.36–0.62)	−1.07 (−1.46)	0.48 (0.38)
7	Natalizumab	42	0.46 (0.34–0.63)	−1.11 (−1.54)	0.46 (0.36)

**Table 2:** Clinical characteristics and prescription details of vedolizumab, ustekinumab, and adalimumab initiators.

Characteristics	Vedolizumab (*n* = 1908)	Adalimumab (*n* = 3706)	Ustekinumab (*n* = 2248)	*p*-Value
Age (years)	40.3 ± 15.6	30.0 ± 14.0	35.2 ± 13.4	<0.001
<20	110 (5.8%)	1029 (27.8%)	136 (6.1%)	
20–39	902 (47.3%)	1771 (47.8%)	1377 (61.3%)	
40–59	620 (32.5%)	777 (21.0%)	584 (26.0%)	
60–79	269 (14.1%)	128 (3.5%)	149 (6.6%)	
≥80	7 (0.4%)	1 (0.0%)	2 (0.1%)	
**Sex**				0.026
Male	1272 (66.7%)	2555 (68.9%)	1586 (70.6%)	
Female	636 (33.3%)	1151 (31.1%)	662 (29.5%)	
Medical specialties of prescriber				<0.001
Gastroenterology	1682 (88.2%)	2558 (69.0%)	1892 (84.2%)	
Other internal medicine	60 (3.1%)	232 (6.3%)	93 (4.1%)	
Others	166 (8.7%)	916 (24.7%)	263 (11.7%)	
Type of IBD				<0.001
Ulcerative colitis	1556 (81.6%)	1370 (37.0%)	917 (40.8%)	
Crohn’s disease	352 (18.5%)	2336 (63.0%)	1331 (59.2%)	
Systemic diseases				
Diabetes mellitus	345 (18.1%)	459 (12.4%)	256 (11.4%)	<0.001
Hypertension	380 (19.9%)	465 (12.6%)	262 (11.7%)	<0.001
Dyslipidemia	428 (22.4%)	617 (16.7%)	368 (16.4%)	<0.001
Liver diseases	1047 (54.9%)	1792 (48.4%)	1110 (49.4%)	<0.001
Chronic kidney diseases	30 (1.6%)	56 (1.5%)	36 (1.6%)	0.960
Extraintestinal manifestations (EIM) at baseline, n (%)				
Musculoskeletal EIMs	530 (27.8%)	845 (22.8%)	556 (24.7%)	<0.001
Dermatologic EIMs	680 (35.6%)	1397 (37.7%)	963 (42.8%)	<0.001
Hepatopancreatobiliary EIMs	371 (19.4%)	559 (15.1%)	452 (20.1%)	<0.001
Ophthalmologic EIMs (other than uveitis)	63 (3.3%)	61 (1.7%)	61 (2.7%)	<0.001
Systemic corticosteroid use at baseline	985 (51.6%)	1578 (42.6%)	1024 (45.6%)	<0.001

Liver diseases were defined by K70-K77. Because these codes are often recorded for reimbursement of liver function testing and hepatitis B screening before and during immunosuppressive therapy, this category may include patients without clinically significant liver disease.

**Table 3:** Incidence rate ratios (IRRs) of anterior and non-anterior uveitis before and after vedolizumab, ustekinumab, and adalimumab initiation.

	Overall Uveitis			Anterior Uveitis			Non-Anterior Uveitis		
Period	Events	IR	IRR (95% CI)	Events	IR	IRR (95% CI)	Events	IR	IRR (95% CI)
Pre-vedolizumab period	28	12.8	Reference	23	10.5	Reference	5	2.3	Reference
Post-vedolizumab period	7	14.7	1.1 (0.5–2.6)	5	10.5	1.0 (0.4–2.6)	2	4.2	1.8 (0.4–9.4)
0–90 days after vedolizumab	0	0.0	N/E	0	0.0	N/E	0	0.0	N/E
>90 days after vedolizumab	7	16.3	1.3 (0.6–2.9)	5	11.6	1.1 (0.4–2.9)	2	4.6	2.0 (0.4–10.5)
Pre-adalimumab period	60	18.6	Reference	55	17.1	Reference	5	1.5	Reference
Post-adalimumab period	29	15.0	0.8 (0.5–1.3)	27	14.0	0.8 (0.5–1.3)	2	1.0	0.7 (0.1–3.5)
0–90 days after adalimumab	4	44.1	2.4 (0.9–6.5)	4	44.1	2.6 (0.9–7.1)	0	0.0	N/E
>90 days after adalimumab	25	13.6	0.7 (0.5–1.2)	23	12.5	0.7 (0.5–1.2)	2	1.1	0.7 (0.1–3.6)
Pre-ustekinumab period	37	14.2	Reference	33	12.7	Reference	4	1.5	Reference
Post-ustekinumab period	4	7.6	0.5 (0.2–1.5)	4	7.6	0.6 (0.2–1.7)	0	0.0	N/E
0–90 days after ustekinumab	0	0.0	N/E	0	0.0	N/E	0	0.0	N/E
>90 days after ustekinumab	4	8.5	0.6 (0.2–1.7)	4	8.5	0.7 (0.2–1.9)	0	0.0	N/E

CI = Confidence interval; IR = Incidence rate per 10,000 person years; IRR = Incidence rate ratio. N/E = Not estimable. Pre-exposure person-time was counted from 1 January 2012. Because patients with uveitis before 1 January 2015, were excluded, no events could occur before 2015; the pre-exposure IRs are therefore likely to be underestimated and the IRRs overestimated, and these comparisons should be regarded as descriptive and exploratory. When no events occurred in a post-exposure window, the IRR is 0 and its 95% CI could not be estimated; this is shown as 0 (N/E).

**Table 4:** Crude and age- and sex-adjusted cumulative incidence (%) of overall uveitis, anterior uveitis, and non-anterior uveitis up to 1 year and up to the end of the study after vedolizumab (Group V), adalimumab (Group A), and ustekinumab (Group U) initiation.

Adverse Event	Cumulative Incidence (%)		Adjusted Incidence (%)	
	1 Year Group V/A/U	Up to Study End Group V/A/U	1 Year Group V/A/U	Up to Study End Group V/A/U
Overall uveitis	0.25/0.17/0.05	0.61/1.29/0.33	0.23/0.15/0.03	0.58/1.14/0.17
Anterior uveitis	0.19/0.17/0.05	0.45/1.16/0.33	0.14/0.15/0.03	0.34/1.03/0.17
Non-anterior uveitis	0.06/<0.01/<0.01	0.16/0.13/<0.01	0.08/<0.01/<0.01	0.23/0.09/<0.01

Adjusted cumulative incidence was directly standardized to the age and sex distribution of the total cohort. Adjusted estimates can exceed crude estimates when events occur in strata that are under-represented in a treatment group relative to the standard population.

**Table 5:** Cox proportional hazards models for overall uveitis in patients with biologic-treated inflammatory bowel disease (IBD).

Variables	Univariable		Multivariable	
	HR (95% CI)	*p*-Value	HR (95% CI)	*p*-Value
Biologics used *		0.420		0.399
Vedolizumab Ustekinumab	0.94 (0.40–2.20) 0.49 (0.17–1.43)		0.67 (0.28–1.62) 0.53 (0.18–1.54)	
Age	1.01 (0.99–1.03)	0.591		
Sex, female ^†^	1.22 (0.64–2.34)	0.545		
IBD, UC ^‡^	2.26 (1.18–4.34)	0.014	1.86 (0.92–3.73)	0.083
Diabetes mellitus	1.12 (0.47–2.67)	0.794		
Hypertension	2.58 (1.31–5.08)	0.006	2.14 (1.07–4.28)	0.031
Dyslipidemia	1.46 (0.72–2.99)	0.298		
Liver diseases	1.57 (0.83–2.97)	0.171		
Chronic kidney diseases	3.16 (0.76–13.08)	0.113		
Baseline corticosteroid use	2.05 (1.08–3.90)	0.028	1.73 (0.90–3.33)	0.100

CI = Confidence interval; UC = Ulcerative colitis. * Adalimumab as reference. ^†^ Male as reference. ^‡^ Crohn’s disease as reference. The multivariable model included biologic group (forced) and covariates with *p* < 0.05 in univariable analyses (IBD subtype, hypertension, and baseline systemic corticosteroid use). Analyses were restricted to 7399 patients without any claim with a uveitis code between 1 January 2015 and the index date.

## Data Availability

The datasets used in this study were obtained from the Health Insurance Review and Assessment Service (HIRA) under a data use agreement. Due to institutional and regulatory restrictions on this nationwide claims database, the data cannot be made publicly available or shared by the authors; researchers may apply for access directly through HIRA’s official data request procedures.

## Supplementary Materials

The following supporting information can be downloaded at: https://www.mdpi.com/article/10.3390/jcm15218291/s1, Figure S1: Flowchart for FAERS disproportionality analysis; Figure S2: Kaplan–Meier curves depicting the time to (A) overall uveitis, (B) anterior uveitis, and (C) non-anterior uveitis in vedolizumab, adalimumab, and ustekinumab initiators with inflammatory bowel disease; Table S1: MedDRA preferred terms for uveitis used in FAERS disproportionality analyses; Table S2: The number of reported infectious uveitis as adverse events for biologics in FAERS database (December 2025); Table S3: The number of reported non-infectious uveitis as adverse events for biologics in FAERS database (December 2025); Table S4: FAERS disproportionality analysis for overall uveitis after excluding cases with uveitis listed as a therapeutic indication. (N = 15,177,486); Table S5: FAERS disproportionality analysis restricted strictly to the single MedDRA preferred term "Uveitis"; Table S6: FAERS disproportionality analysis restricted strictly to reports with confirmed inflammatory bowel disease (Crohn’s disease or ulcerative colitis) indications. (N = 368,256); Table S7: Diagnostic and procedural codes used to identify ocular trauma and intraocular procedures; Table S8: Drugs whose users were excluded due to reported associations between these drugs and drug-induced uveitis; Table S9: Definitions and Korean Classification of Disease codes Used to Define types of Uveitis; Table S10: Incidence rate ratios (IRRs) of anterior vs non-anterior uveitis uveitis before and after vedolizumab, ustekinumab, and adalimumab initiation in patients with ulcerative colitis (N = 3843); Table S11: Incidence rate ratios (IRRs) of anterior vs non-anterior uveitis uveitis before and after vedolizumab, ustekinumab, and adalimumab initiation in patients with Crohn’s disease (N = 4019); Table S12: Incidence rate ratios of overall uveitis by treatment period in switched groups from adalimumab to vedolizumab or ustekinumab and comparison of post-exposure risk between vedolizumab and ustekinumab.

## References

[1] Rogler G., Singh A., Kavanaugh A., Rubin D.T. (2021). Extraintestinal Manifestations of Inflammatory Bowel Disease: Current Concepts, Treatment, and Implications for Disease Management. Gastroenterology.

[2] Ramos-Casals M., Roberto Perez A., Diaz-Lagares C., Cuadrado M.J., Khamashta M.A., BS Group (2010). Autoimmune diseases induced by biological agents: A double-edged sword?. Autoimmun. Rev..

[3] Kremenevski I., Sander O., Sticherling M., Raithel M. (2022). Paradoxical Reactions to Biologicals in Chronic Inflammatory Systemic Diseases. Dtsch. Arztebl. Int..

[4] Harbord M., Annese V., Vavricka S.R., Allez M., Barreiro-de Acosta M., Boberg K.M., Burisch J., De Vos M., De Vries A.M., Dick A.D. (2016). The First European Evidence-based Consensus on Extra-intestinal Manifestations in Inflammatory Bowel Disease. J. Crohns Colitis.

[5] Suttorp-Schulten M.S., Rothova A. (1996). The possible impact of uveitis in blindness: A literature survey. Br. J. Ophthalmol..

[6] Shah J., Shah A., Hassman L., Gutierrez A. (2021). Ocular Manifestations of Inflammatory Bowel Disease. Inflamm. Bowel Dis..

[7] Feagan B.G., Rutgeerts P., Sands B.E., Hanauer S., Colombel J.F., Sandborn W.J., Van Assche G., Axler J., Kim H.J., Danese S. (2013). Vedolizumab as induction and maintenance therapy for ulcerative colitis. N. Engl. J. Med..

[8] Sandborn W.J., Feagan B.G., Rutgeerts P., Hanauer S., Colombel J.F., Sands B.E., Lukas M., Fedorak R.N., Lee S., Bressler B. (2013). Vedolizumab as induction and maintenance therapy for Crohn’s disease. N. Engl. J. Med..

[9] Jaffe G.J., Dick A.D., Brézin A.P., Nguyen Q.D., Thorne J.E., Kestelyn P., Barisani-Asenbauer T., Franco P., Heiligenhaus A., Scales D. (2016). Adalimumab in Patients with Active Noninfectious Uveitis. N. Engl. J. Med..

[10] Nguyen Q.D., Merrill P.T., Jaffe G.J., Dick A.D., Kurup S.K., Sheppard J., Schlaen A., Pavesio C., Cimino L., Van Calster J. (2016). Adalimumab for Prevention of Uveitic Flare in Patients with Inactive Non-Infectious Uveitis Controlled by Corticosteroids (VISUAL II): A Multicentre, Double-Masked, Randomised, Placebo-Controlled Phase 3 Trial. Lancet.

[11] Feagan B.G., Sandborn W.J., Gasink C., Jacobstein D., Lang Y., Friedman J.R., Blank M.A., Johanns J., Gao L.L., Miao Y. (2016). Ustekinumab as Induction and Maintenance Therapy for Crohn’s Disease. N. Engl. J. Med..

[12] Wyant T., Fedyk E., Abhyankar B. (2016). An Overview of the Mechanism of Action of the Monoclonal Antibody Vedolizumab. J. Crohns Colitis.

[13] Rogler G. (2019). Mechanism of action of vedolizumab: Do we really understand it?. Gut.

[14] Diaz L.I., Keihanian T., Schwartz I., Bin Kim S., Calmet F., Alejandra Quintero M., Abreu M.T. (2020). Vedolizumab-Induced De Novo Extraintestinal Manifestations. Gastroenterol. Hepatol..

[15] Chateau T., Bonovas S., Le Berre C., Mathieu N., Danese S., Peyrin-Biroulet L. (2019). Vedolizumab Treatment in Extra-Intestinal Manifestations in Inflammatory Bowel Disease: A Systematic Review. J. Crohns Colitis.

[16] Fridman G., LaMattina K.C. (2021). New-onset anterior uveitis in two patients on ustekinumab for inflammatory bowel disease. Indian J. Ophthalmol. Case Rep..

[17] Godwin K.S., Mathai M., Do B.K. (2022). A case of ustekinumab-induced sclerouveitis. Am. J. Ophthalmol. Case Rep..

[18] Toussirot E., Aubin F. (2016). Paradoxical reactions under TNF-alpha blocking agents and other biological agents given for chronic immune-mediated diseases: An analytical and comprehensive overview. RMD Open.

[19] Xu S., Song Z., Li Y., Bai J., Wang D., Wang E., Wang J. (2025). Drug-induced immune-mediated uveitis: A real-world pharmacovigilance analysis using FAERS and JADER databases. BMJ Open Ophthalmol..

[20] Cutroneo P.M., Sartori D., Tuccori M., Crisafulli S., Battini V., Carnovale C., Rafaniello C., Capuano A., Poluzzi E., Moretti U. (2023). Conducting and interpreting disproportionality analyses derived from spontaneous reporting systems. Front. Drug Saf. Regul..

[21] Hauben M., Zhou X. (2003). Quantitative methods in pharmacovigilance: Focus on signal detection. Drug Saf..

[22] Kim J., Ahn S.J., Park J., Gower E.W., Chung J.E. (2025). Systemic Drugs Associated with Maculopathy. JAMA Ophthalmol..

[23] Evans S.J., Waller P.C., Davis S. (2001). Use of proportional reporting ratios (PRRs) for signal generation from spontaneous adverse drug reaction reports. Pharmacoepidemiol. Drug Saf..

[24] Hwang S., Kim J., Chung J.E., Ahn S.J. (2026). Maculopathy in Paclitaxel Users: Nationwide Drug Use, Incidence, and Risk Factors. Ophthalmol. Retin..

[25] Kim J., Chung J.E., Ahn S.J. (2025). Nationwide health claims analysis of phenothiazine-associated retinopathy risk in chlorpromazine and perphenazine users. Sci. Rep..

[26] Kim J.A., Yoon S., Kim L.Y., Kim D.S. (2017). Towards Actualizing the Value Potential of Korea Health Insurance Review and Assessment (HIRA) Data as a Resource for Health Research: Strengths, Limitations, Applications, and Strategies for Optimal Use of HIRA Data. J. Korean Med. Sci..

[27] Song S.O., Jung C.H., Song Y.D., Park C.Y., Kwon H.S., Cha B.S., Park J.Y., Lee K.U., Ko K.S., Lee B.W. (2014). Background and data configuration process of a nationwide population-based study using the Korean national health insurance system. Diabetes Metab. J..

[28] Kim J., Kwon H.Y., Ahn S.J. (2024). COVID-19 Vaccine-Associated Uveitis in Patients with a History of Uveitis. JAMA Ophthalmol..

[29] Rim T.H., Kim S.S., Ham D.I., Yu S.Y., Chung E.J., Lee S.C., Korean Uveitis Society (2018). Incidence and prevalence of uveitis in South Korea: A nationwide cohort study. Br. J. Ophthalmol..

[30] Dubinsky M.C., Cross R.K., Sandborn W.J., Long M., Song X., Shi N., Ding Y., Eichner S., Pappalardo B., Ganguli A. (2018). Extraintestinal Manifestations in Vedolizumab and Anti-TNF-Treated Patients with Inflammatory Bowel Disease. Inflamm. Bowel Dis..

[31] Lin H., Zhang J., Liang C., Wu D., Tan Y., Luo L., Liu Z. (2024). Differences in the prevalence of uveitis between Crohn’s disease and ulcerative colitis: A systematic review and meta-analysis. Acta Ophthalmol..

