# Supplementary material for: Uveitis in Patients with Inflammatory Bowel Disease: Pharmacovigilance Signal Detection and Nationwide Claims-Based Evaluation
Source: J Clin Med. 2026 Oct 24;15(21):8291. doi: 10.3390/jcm15218291 (PMC13763909; doi:10.3390/jcm15218291)

Supplementary Table S1. MedDRA preferred terms for uveitis used in FAERS disproportionality analyses

Preferred Terms	Infectious	Non-infectious	Narrow definitions
Uveitis	✓	✓	✓
Autoimmune uveitis		✓	✓
Anterior chamber inflammation	✓	✓	✓
Enteropathic uveitis		✓	✓
Eye inflammation	✓	✓	
Immune-mediated uveitis		✓	✓
Infective uveitis	✓		✓
Keratouveitis	✓	✓	✓
Tubulointerstitial nephritis and uveitis syndrome		✓	✓
Uveitis-glaucoma-hyphaema syndrome	✓	✓	✓
Viral keratouveitis	✓		✓
Viral uveitis	✓		✓
Behcet's syndrome		✓	
Iridocyclitis	✓	✓	✓
Non-infective chorioretinitis		✓	✓
Chorioretinitis	✓	✓	✓
Scleritis	✓	✓	
Sympathetic ophthalmia		✓	✓
Eye infection syphilitic	✓		✓

Supplementary Table S2. The number of reported infectious uveitis as adverse events for biologics in FAERS database (December 2025)

Medication	No. of reports	ROR (95% CI)	IC(IC025)	EBGM(EBGM05)
Adalimumab	2791	4.9 (4.76-5.16)	2.14 (2.09)	4.42 (4.28)
Infliximab	1781	12 (11.43-12.6)	3.45 (3.38)	10.93 (10.5)
Vedolizumab	286	2.96 (2.64-3.33)	1.55 (1.38)	2.93 (2.65)
Golimumab	197	3.2 (2.78-3.69)	1.67 (1.46)	3.17 (2.82)
Ustekinumab	64	0.65 (0.51-0.83)	-0.62 (-0.97)	0.65 (0.53)
Risankizumab	52	0.51 (0.39-0.66)	-0.98 (-1.37)	0.51 (0.4)
Natalizumab	41	0.48 (0.35-0.65)	-1.05 (-1.49)	0.48 (0.37)

Supplementary Table S3. The number of reported non-infectious uveitis as adverse events for biologics in FAERS database (December 2025)

Medication	No. of reports	ROR (95% CI)	IC(IC025)	EBGM(EBGM05)
Adalimumab	2984	5 (4.81-5.2)	2.16 (2.1)	4.45 (4.31)
Infliximab	1894	12.04 (11.48-12.63)	3.45 (3.39)	10.96 (10.53)
Vedolizumab	288	2.81 (2.5-3.16)	1.47 (1.31)	2.78 (2.52)
Golimumab	204	3.13 (2.72-3.59)	1.63 (1.43)	3.1 (2.76)
Ustekinumab	64	0.61 (0.48-0.78)	-0.7 (-1.06)	0.61 (0.5)
Risankizumab	52	0.48 (0.36-0.63)	-1.06 (-1.46)	0.48 (0.38)
Natalizumab	41	0.45 (0.33-0.62)	-1.13 (-1.58)	0.46 (0.35)

Supplementary Table S4. FAERS disproportionality analysis for overall uveitis after excluding cases with uveitis listed as a therapeutic indication. (N = 15,177,486.)

Medication	No. of reports	ROR (95% CI)	IC(IC025)	EBGM(EBGM05)
Adalimumab	1875	3.42 (3.26-3.59)	1.67 (1.61)	3.19 (3.06)
Infliximab	1610	11.53 (10.96-12.14)	3.4 (3.33)	10.56 (10.12)
Vedolizumab	285	3.12 (2.78-3.51)	1.62 (1.46)	3.08 (2.79)
Golimumab	198	3.41 (2.96-3.92)	1.75 (1.55)	3.37 (3)
Ustekinumab	63	0.68 (0.53-0.87)	-0.56 (-0.92)	0.68 (0.55)
Risankizumab	51	0.52 (0.4-0.69)	-0.93 (-1.32)	0.53 (0.42)
Natalizumab	42	0.52 (0.39-0.71)	-0.94 (-1.37)	0.52 (0.41)

Supplementary Table S5. FAERS disproportionality analysis restricted strictly to the single MedDRA preferred term "Uveitis"

Medication	No. of reports	ROR (95% CI)	IC(IC025)	EBGM(EBGM05)
Adalimumab	1861	7.05 (6.7-7.41)	2.58 (2.51)	5.97 (5.72)
Infliximab	1578	23.16 (21.95-24.44)	4.29 (4.22)	19.62 (18.76)
Vedolizumab	190	4.00 (3.46-4.62)	1.98 (1.77)	3.94 (3.49)
Golimumab	151	5.00 (4.25-5.87)	2.30 (2.07)	4.93 (4.31)
Ustekinumab	46	0.95 (0.71-1.27)	-0.08 (-0.49)	0.95 (0.74)
Risankizumab	17	0.33 (0.21-0.54)	-1.57 (-2.26)	0.34 (0.23)
Natalizumab	23	0.55 (0.36-0.82)	-0.87 (-1.45)	0.55 (0.39)

Supplementary Table S6. FAERS disproportionality analysis restricted strictly to reports with confirmed inflammatory bowel disease (Crohn's disease or ulcerative colitis) indications. (N=368,256)

Medication	No. of reports	ROR (95% CI)	IC(IC025)	EBGM(EBGM05)
Adalimumab	402	0.41 (0.37-0.46)	-0.94 (-1.08)	0.52 (0.48)
Infliximab	1250	5.87 (5.39-6.4)	1.60 (1.51)	3.02 (2.81)
Vedolizumab	282	0.76 (0.67-0.86)	-0.34 (-0.5)	0.79 (0.71)
Golimumab	6	0.27 (0.12-0.6)	-1.86 (-3.01)	0.28 (0.14)
Ustekinumab	36	0.24 (0.17-0.33)	-1.99 (-2.46)	0.25 (0.19)
Risankizumab	19	0.19 (0.12-0.29)	-2.36 (-3)	0.2 (0.13)
Natalizumab	0	0	-Inf	0

Supplementary Table S7. Diagnostic and procedural codes used to identify ocular trauma and intraocular procedures

Category	KCD-8 codes	Conditions
Ocular Trauma	S05.0	Injury of conjunctiva and corneal abrasion without foreign body
	S05.1	Contusion of eyeball and orbital tissues
	S05.2	Ocular laceration and rupture with prolapse or loss of intraocular tissue
	S05.3	Ocular laceration without prolapse or loss of intraocular tissue
	S05.4	Penetrating wound of orbit with or without foreign body
	S05.5	Penetrating wound with foreign body of eyeball
	S05.6	Penetrating wound without foreign body of eyeball
	S05.7	Avulsion of eye
	S05.8	Other injuries of eye and orbit
	S05.9	Unspecified injury of eye and orbit
Intraocular Procedures	S4891, S4892	Removal of Intraocular Foreign Body
	S4911	Implantation of Ocular Implant
	S5021, S5030, S5035	Synechiolysis/ Optical Iridectomy/ Suture Repairing of Iris, Ciliary Body
	S5033, S5039, S5040, S5041, S5042, S5043, S5044, S5045, S5047, S5048, S5049	Surgery for Glaucoma
	S5050	Vitreous Aspiration
	S5070	Intravitreal Injection
	S5071, S5072	Resection of Vitreous Band (Anterior portion/ Posterior portion)
	S5080, S5091	Anterior Chamber Paracentesis/ Anterior Chamber Irrigation
	S5110, S5111, S5112, S5116, S5117, S5118, S5119	Surgery for Cataract or Lens, Intraocular Lens Implantation or Exchange
	S5121, S5122, S5145	Vitrectomy, Periretinal Membrane Peeling
	S5371, S5372, S5374, S5380, S5381	Keratoplasty, Primary Closure of Cornea

Supplementary Table S8. Drugs whose users were excluded due to reported associations between these drugs and drug-induced uveitis

Category	Component name	Component code
Bisphosphonates	Sodium alendronate	17484, 17486, 17488, 17511, 17664
	Risedronate sodium	17592, 17634, 17636
	Pamidronate disodium	24811
	Ibandronate sodium monohydrate	17666
	Zoledronic acid	24824, 24827, 24829
Rifabutin	Rifabutin	18405
Immune checkpoint inhibitors	Nivolumab	25381, 25383, 25385
	Pembrolizumab	25387
	Atezolizumab	25403
	Ipilimumab	25377, 25379

Supplementary Table S9. Definitions and Korean Classification of Disease codes Used to Define types of Uveitis

		ICD-10 codes	KCD-8 codes	Conditions
Infectious Uveitis	Anterior	H22.0	H22.0	Iridocyclitis in infectious and parasitic diseases cla ssified elsewhere<br>Iridocylitis in gonococcal infection<br>Iridocylitis in herpesviral [herpes simplex] infection<br>Iridocylitis in syphilis(secondary)<br>Iridocylitis in tuberculosis<br>Iridocylitis in zoster
	Posterior	H32.0	H32.0	Chorioretinal inflammation in infectious and parasiti c diseases classified elsewhere<br>Syphilitic, late chorioretinitis<br>Toxoplasma chorioretinitis<br>Tuberculous chorioretinitis
		H44.1		* In the KCD-8 code,<br>H44.1 represents endophthalmitis (not panuveitis)
Non-infectious Uveitis	Anterior	H20.0	H20.0	Acute and subacute iridocyclitis
		H20.1	H20.1	Chronic iridocyclitis
		H20.2	H20.2	Lens-induced iridocylitis
		H20.8	H20.8	Other iridocyclitis
		H20.9	H20.9	Iridocyclitis, unspecified
		H22.1	H22.1	Iridocyclitis in other diseases classified elsewhere<br>Iridocyclitis in ankylosing spondylitis<br>Iridocyclitis in sarcoidosis
		H22.8	H22.8	Other disorders of iris and ciliary body in diseases classified elsewhere
	Non-anterior	H30.0	H30.0	Focal chorioretinal inflammation<br>Focal chorioretinitis<br>Focal choroiditis<br>Focal retinitis<br>Focal retinochoroiditis
		H30.1	H30.1	Disseminated chorioretinal inflammation<br>Disseminated chorioretinitis<br>Disseminated choroiditis<br>Disseminated retinitis<br>Disseminated retinochoroiditis
		H30.2	H30.2	Posterior cyclitis<br>Pars planitis
		H30.8	H30.8	Other chorioretinal inflammations<br>Harada's disease
		H30.9	H30.9	Chorioretinal inflammation, unspecified<br>Chorioretinitis NOS<br>Choroiditis NOS<br>Retinitis NOS<br>Retinochoroiditis NOS
		H35.06	H35.05	Retinal vasculitis<br>Retinal perivasculitis

Supplementary Table S10. Incidence rate ratios (IRRs) of anterior vs non-anterior uveitis uveitis before and after vedolizumab, ustekinumab, and adalimumab initiation in patients with ulcerative colitis (N=3,843)

**Period**	**Overall uveitis**			**Anterior uveitis**			**Non-anterior uveitis**		
	Events	IR	IRR (95% CI)	Events	IR	IRR (95% CI)	Events	IR	IRR (95% CI)
Pre-vedolizumab period	27	15.2	Reference	22	12.4	Reference	5	2.8	Reference
Post-vedolizumab period	7	17.7	1.2 (0.5-2.7)	5	12.6	1.0 (0.4-2.7)	2	5.0	1.8 (0.4-9.3)
0-90 days after vedolizumab	0	0.0	N/E	0	0.0	N/E	0	0.0	N/E
>90 days after vedolizumab	7	19.7	1.3 (0.6-3.0)	5	14.0	1.1 (0.4-3.0)	2	5.6	2.0 (0.4-10.3)
Pre-adalimumab period	28	24.5	Reference	27	23.6	Reference	1	0.9	Reference
Post-adalimumab period	17	22.4	0.9 (0.5-1.7)	16	21.1	0.9 (0.5-1.7)	1	1.3	1.5 (0.1-24.1)
0-90 days after Adalimumab	3	89.6	3.7 (1.1-12.0)	3	89.6	3.8 (1.2-12.5)	0	0.0	N/E
>90 days after Adalimumab	14	19.5	0.8 (0.4-1.5)	13	18.0	0.8 (0.4-1.5)	1	1.4	1.6 (0.1-25.3)
Pre-ustekinumab period	19	17.3	Reference	17	15.4	Reference	2	1.8	Reference
Post-ustekinumab period	2	11.6	0.7 (0.2-2.9)	2	11.6	0.8 (0.2-3.3)	0	0.0	N/E
0-90 days after Ustekinumab	0	0.0	N/E	0	0.0	N/E	0	0.0	N/E
>90 days after Ustekinumab	2	13.3	0.8 (0.2-3.3)	2	13.3	0.9 (0.2-3.7)	0	0.0	N/E

CI = confidence interval; IR = Incidence rate per 10,000 person-years; IRR = Incidence rate ratio; N/E = Not estimable. Pre-exposure person-time was counted from January 1, 2012. Because patients with uveitis before January 1, 2015, were excluded, no events could occur before 2015; the pre-exposure IRs are therefore likely to be underestimated and the IRRs overestimated, and these comparisons should be regarded as descriptive and exploratory. When no events occurred in a post-exposure window, the IRR is 0 and its 95% CI could not be estimated; this is shown as 0 (NE).

Supplementary Table S11. Incidence rate ratios (IRRs) of anterior vs non-anterior uveitis uveitis before and after vedolizumab, ustekinumab, and adalimumab initiation in patients with Crohn's disease (N=4,019)

**Period**	**Overall uveitis**			**Anterior uveitis**			**Non-anterior uveitis**		
	Events	IR	IRR (95% CI)	Events	IR	IRR (95% CI)	Events	IR	IRR (95% CI)
Pre-vedolizumab period	1	2.4	Reference	1	2.4	Reference	0	0.0	Reference
Post-vedolizumab period	0	0.0	N/E	0	0.0	N/E	0	0.0	N/E
0-90 days after vedolizumab	0	0.0	N/E	0	0.0	N/E	0	0.0	N/E
>90 days after vedolizumab	0	0.0	N/E	0	0.0	N/E	0	0.0	N/E
Pre-adalimumab period	32	15.4	Reference	28	13.5	Reference	4	1.9	Reference
Post-adalimumab period	12	10.2	0.7 (0.3-1.3)	11	9.4	0.7 (0.3-1.4)	1	0.8	0.4 (0.1-3.9)
0-90 days after adalimumab	1	17.5	1.1 (0.2-8.3)	1	17.5	1.3 (0.2-9.6)	0	0.0	N/E
>90 days after adalimumab	11	9.9	0.6 (0.3-1.3)	10	8.9	0.7 (0.3-1.4)	1	0.9	N/E
Pre-ustekinumab period	18	11.9	Reference	16	10.6	Reference	2	1.3	Reference
Post-ustekinumab period	2	5.6	0.5 (0.1-2.0)	2	5.6	0.5 (0.1-2.3)	0	0.0	N/E
0-90 days after Ustekinumab	0	0.0	N/E	0	0.0	N/E	0	0.0	N/E
>90 days after Ustekinumab	2	6.2	0.5 (0.1-2.2)	2	6.2	0.6 (0.1-2.5)	0	0.0	N/E

CI = confidence interval; IR = Incidence rate per 10,000 person-years; IRR = Incidence rate ratio; N/E = Not estimable. Pre-exposure person-time was counted from January 1, 2012. Because patients with uveitis before January 1, 2015, were excluded, no events could occur before 2015; the pre-exposure IRs are therefore likely to be underestimated and the IRRs overestimated, and these comparisons should be regarded as descriptive and exploratory. When no events occurred in a post-exposure window, the IRR is 0 and its 95% CI could not be estimated; this is shown as 0 (NE).

Supplementary Table S12 Incidence rate ratios of overall uveitis by treatment period in switched groups from adalimumab to vedolizumab or ustekinumab and comparison of post-exposure risk between vedolizumab and ustekinumab

Group	Adalimumab period		Vedolizumab or Ustekinumab period			Vedolizumab vs. ustekinumab
	Events	Incidence rate per 10000 PY	Events	Incidence rate per 10000 PY	IRR (95% CI)	HR (95% CI)
Adalimumab to vedolizumab	0	0	2	18.9	N/E	Ref
Adalimumab to ustekinumab	2	13.1	3	24.2	1.9 (0.3-11.1)	0.88 (0.14-5.58)

CI = confidence interval; IRR = incidence rate ratio; N/E = not estimable (incalculable due to a zero denominator); PY = person-years.

* In the adalimumab-to-vedolizumab group, no events occurred during the adalimumab period (person-time > 0; IR, 0.0 per 10,000 PYs); the IRR could not be estimated because no events occurred in the reference period.

Supplementary Figure S1. Flowchart for FAERS disproportionality analysis.

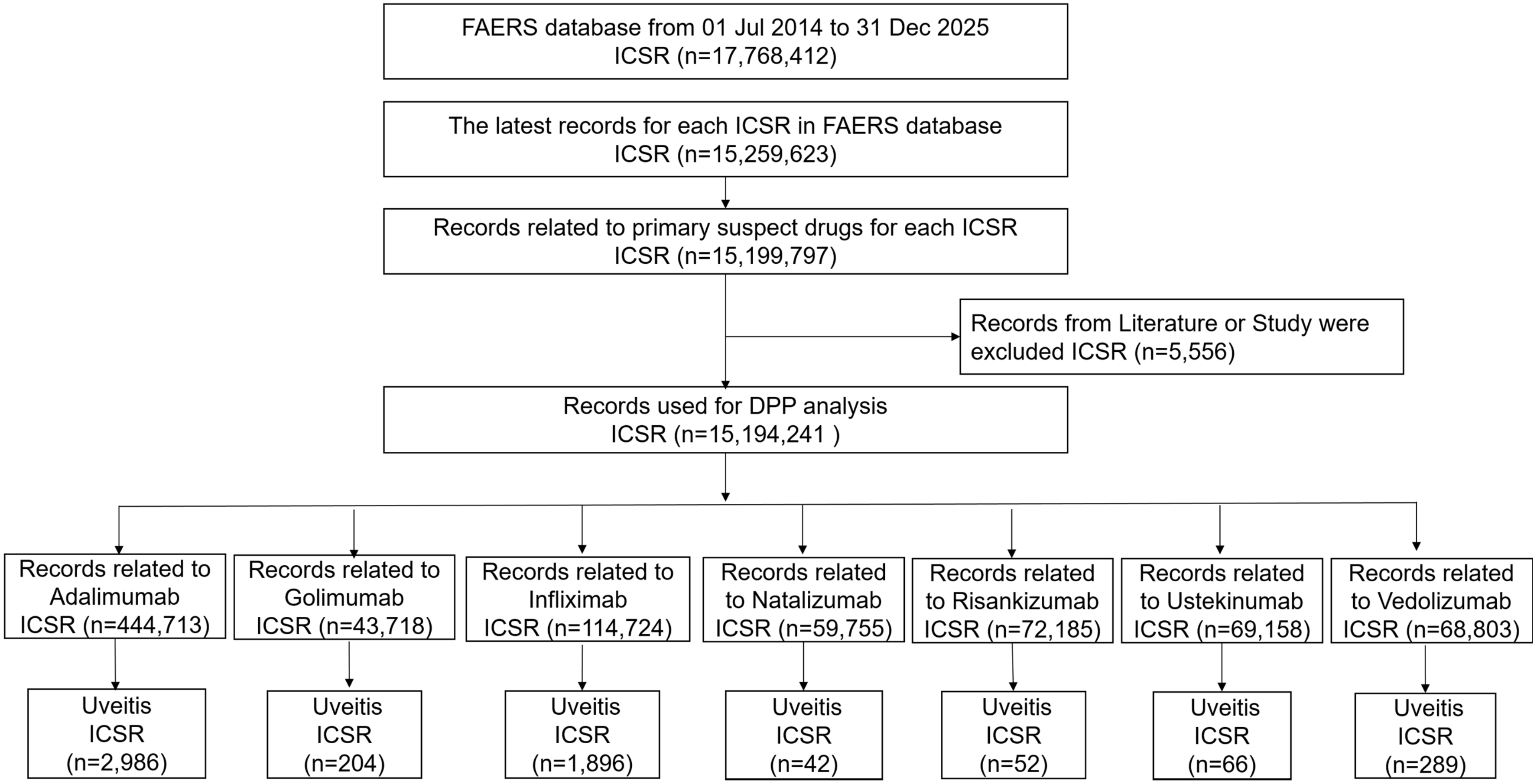

Supplementary Figure S2. Kaplan–Meier curves depicting the time to (A) overall uveitis, (B) anterior uveitis, and (C) non-anterior uveitis in vedolizumab, adalimumab, and ustekinumab initiators with inflammatory bowel disease.

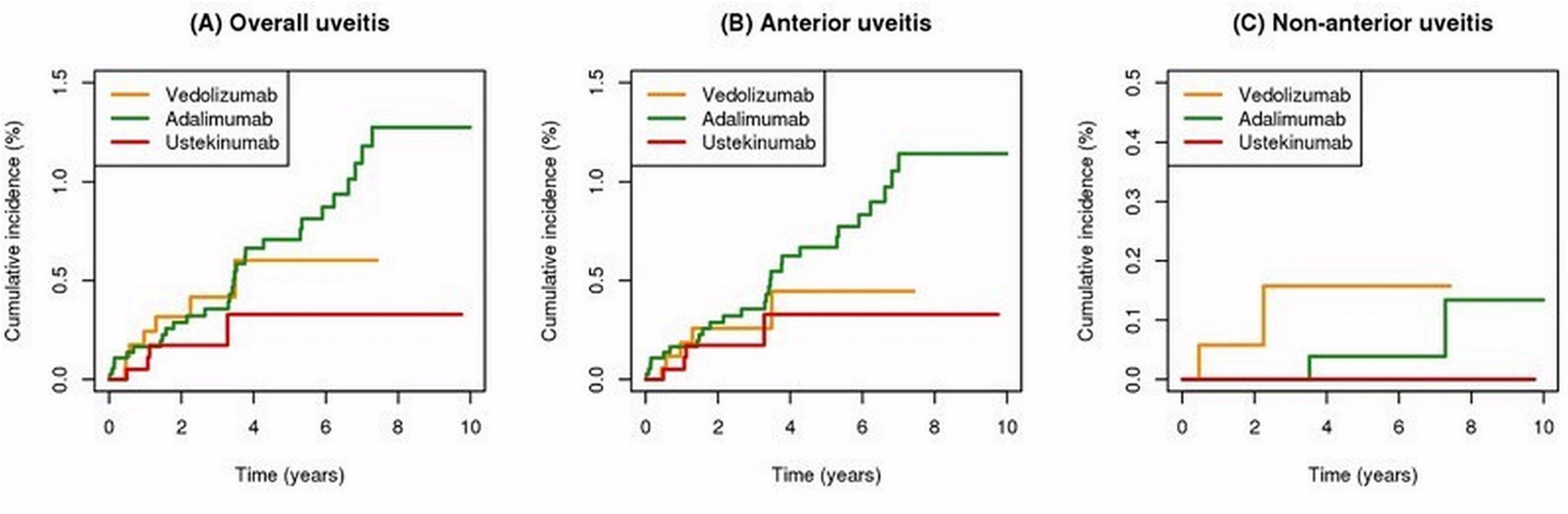

Supplement: Supplementary file 1 [file jcm-15-08291-s001.zip › jcm-4570506-supplementary.pdf]
